# Intra‐season variations in distribution and abundance of humpback whales in the West Antarctic Peninsula using cruise vessels as opportunistic platforms

**DOI:** 10.1002/ece3.8571

**Published:** 2022-02-09

**Authors:** John Elling Deehr Johannessen, Martin Biuw, Ulf Lindstrøm, Victoria Marja Sofia Ollus, Lucía Martina Martín López, Kalliopi C. Gkikopoulou, Wessel Chris Oosthuizen, Andrew Lowther

**Affiliations:** ^1^ Department of Arctic Biology The Arctic University of Tromsø Tromsø Norway; ^2^ 8016 Norwegian Polar Institute Tromsø Norway; ^3^ Institute of Marine Research Tromsø Norway; ^4^ School of Environmental Sciences University of Liverpool Liverpool UK; ^5^ Sea Mammal Research Unit School of Biology Scottish Ocean Institute University of St Andrews St Andrews UK; ^6^ University of Cape Town Cape Town South Africa

**Keywords:** Antarctic Peninsula, density surface modeling, ecosystem interactions, humpback whale, platforms of opportunity, spatiotemporal variation

## Abstract

Fine‐scale knowledge of spatiotemporal dynamics in cetacean distribution and abundance throughout the Western Antarctic Peninsula (WAP) is sparse yet essential for effective ecosystem‐based management (EBM). Cruise vessels were used as platforms of opportunity to collect data on the distribution and abundance of humpback whales (*Megaptera novaeangliae*) during the austral summer of 2019/2020 in a region that is also important for the Antarctic krill (*Euphausia superba*) fishery, to assess potential spatiotemporal interactions for future use in EBM. Data were analyzed using traditional design‐based line transect methodology and spatial density surface hurdle models fitted using a set of physical environmental covariates to estimate the abundance and distribution of whales in the area, and to describe their temporal dynamics. Our results indicate a rapid increase in humpback whale abundance in the Bransfield and Gerlache Straits through December, reaching a stable abundance by mid‐January. The distribution of humpback whales appeared to change from a patchier distribution in the northern Gerlache Strait to a significantly concentrated presence in the central Gerlache and southern Bransfield Straits, followed by a subsequent dispersion throughout the area. Abundance estimates agreed well with previous literature, increasing from approximately 7000 individuals in 2000 to a peak of 19,107 in 2020. Based on these estimates, we project a total krill consumption of between 1.4 and 3.7 million tons based on traditional and contemporary literature on per capita krill consumption of whales, respectively. When taken in the context of krill fishery catch data in the study area, we conclude that there is minimal spatiotemporal overlap between humpback whales and fishery activity during our study period of November–January. However, there is potential for significant interaction between the two later in the feeding season, but cetacean survey efforts need to be extended into late season in order to fully characterize this potential overlap.

## Graphical abstract

Fine‐scale knowledge of spatiotemporal dynamics in cetacean distribution and abundance throughout the Western Antarctic Peninsula (WAP) is sparse yet essential for effective ecosystem‐based management (EBM). Our results indicate a rapid increase in humpback whale abundance in the Bransfield and Gerlache Straits through December, reaching a stable abundance by mid‐January, while their distribution appeared to change from a patchier distribution to a significantly concentrated presence in the northern Gerlache and southern Bransfield Straits, followed by a subsequent dispersion throughout the study area. These trends may lead to interactions with other ecosystem elements such as breeding chinstrap penguins and anthropogenic fishery vessels.

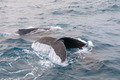

## 1 INTRODUCTION

As a result of overexploitation, fishing has shifted focus to areas beyond national jurisdictions (Kawaguchi & Nicol, 2007; Nicol et al., 2012). The fishery for Antarctic krill (*Euphausia superba*, henceforth krill) is the largest fishery in the Southern Ocean in terms of biomass (Nicol et al., 2012). Over the preceding three decades, its spatial footprint has contracted around the Western Antarctic Peninsula (WAP; Krüger, 2019; Santa Cruz et al., 2018), and in combination with a steady increase in catch levels that now represent the highest the fishery has ever seen, concerns about fisheries impacts on the broader marine ecosystem are being raised.

**FIGURE 1:**
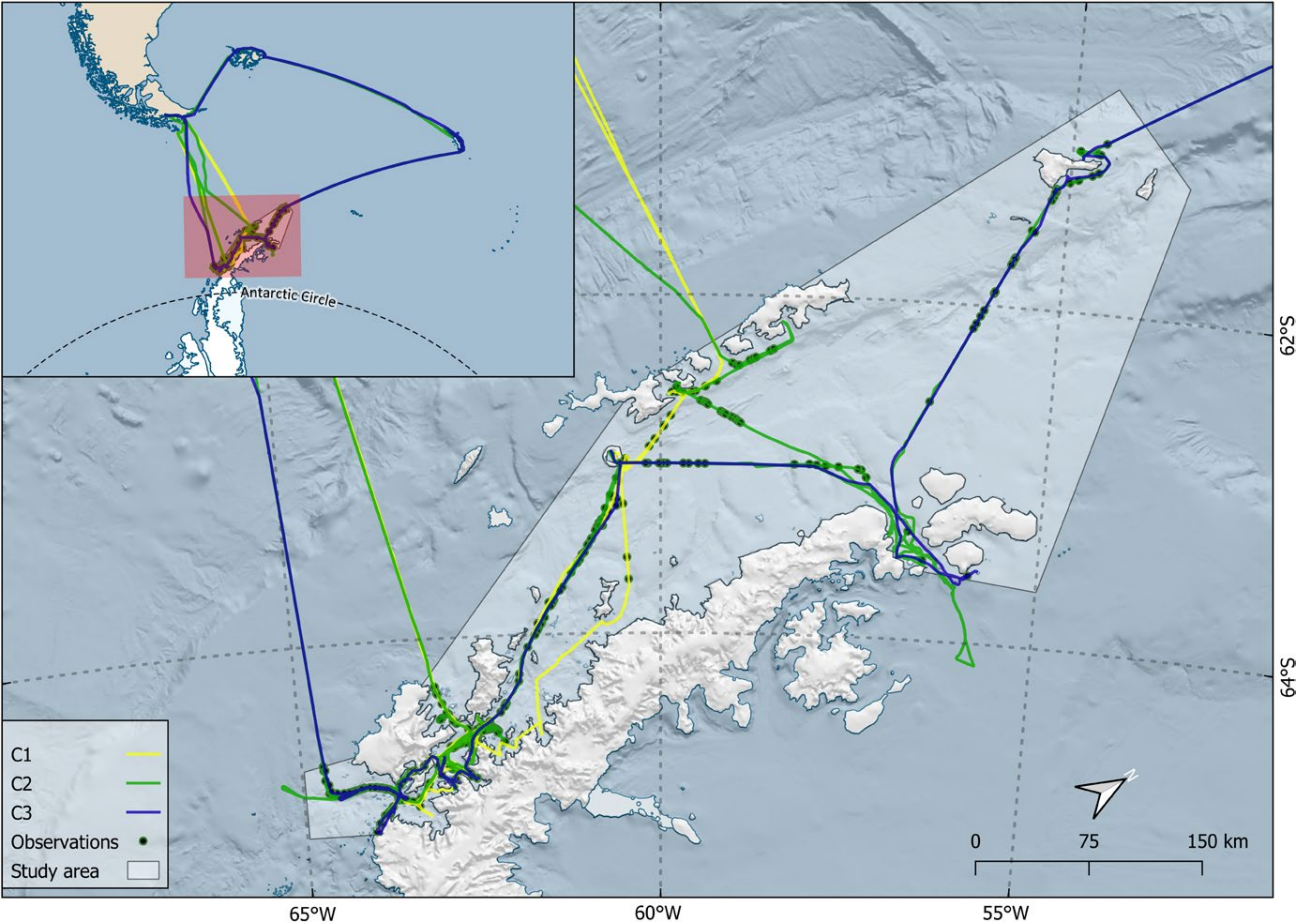
The study area for analysis, marked white, survey transects for the different cruises (C1 (25.11.19–12.12.19), C2 (16–27.12.29), and C3 (12–18.01.20)) and humpback whale observations (black dots) from all cruises. The Bransfield and Gerlache (indicated by arrow) Straits and Drake Passage are shown. Inlet map shows study area and tracklines in relation to the South American and Antarctic continents. Map produced using Quantarctica (Matsuoka et al., 2021)

Currently, the fishery is managed by the Commission for the Conservation of Antarctic Marine Living Resources (CCAMLR) using a precautionary catch limit of 620,000 tons, distributed between subareas across the Drake Passage, Antarctic Peninsula, and Scotia Sea (Figure 1; Hill et al., 2016). Additional quotas are available in East Antarctic waters; however, the fishery has not operated there at commercially meaningful levels for several decades (CCAMLR Secretariat, 2021). Despite the fishery operating almost exclusively in CCAMLR management Area 48 (covering the WAP and Scotia Sea) since the turn of the millennia, two large‐scale krill surveys in this area conducted two decades apart showed remarkably similar biomass estimates (ca. 60 million tons; Macaulay et al., 2019). Thus, given that the fishery currently takes less than 0.75% of the estimated biomass in area 48, there is increasing interest in expanding the quota beyond the current trigger level (Nicol et al., 2012).

In addition to pressures from fishing, rapid climate warming (Ducklow et al., 2013; Meredith & King, 2005) may potentially affect the spatial distribution, abundance, and recruitment of krill (Atkinson et al., 2004, 2019), which in turn impact krill‐dependent predators in the WAP area (Krüger et al., 2020; Watters et al., 2020). The interactive effects of climate‐driven modification of the WAP on krill dynamics and various population trajectories of upper trophic predators are likely to be complex (Lynch et al., 2012; Trivelpiece et al., 2011). In light of this, CCAMLR is currently looking to implement ecosystem‐based management (EBM) to adaptively manage catch limits and spatial allocations in a manner that avoids negatively impacting krill‐dependent aspects of the ecosystem (SC‐CAMLR, 2011). Implicit to this is the need for ecological information on krill‐consuming predators.

For more than two centuries, pinnipeds and large cetaceans were subjected to uncontrolled harvesting (Trathan & Reid, 2009), with some species driven to near extinction (Tulloch et al., 2018). Surma et al. (2014) estimated that this reduction of large‐bodied krill predators in the ecosystem may have resulted in the competitive release of krill, *sensu* the Krill Surplus Hypothesis (Laws et al., 1977). Under this hypothesis, the standing stock of krill is estimated to have increased approximately 17%–25% in response to declines in krill predators such as seals and whales, allowing population increases of less competitive krill predators with shorter generation times such as penguins (particularly gentoo *Pygoscelis papua*, adélie *P*. *adeliae*, and chinstrap penguins *P*. *antarcticus*) and pinnipeds (particularly crabeater seals *Lobodon carcinophagus* and Antarctic fur seals *Arctocephalus gazella*; Surma et al., 2014). Following the moratorium prohibiting the commercial catch of whales, set by the International Whaling Committee in 1986, many cetacean populations are showing signs of recovering toward their preharvest levels (Bettridge et al., 2015; Thomas et al., 2016; Tulloch et al., 2018; Zerbini et al., 2019). Consequently, krill consumption by cetaceans in the CCAMLR Area 48 is likely to have increased in the postwhaling era in line with rebounding populations. The krill consumption by cetaceans in area 48 during the austral summer season of 1999/2000 was estimated to 1.6–2.7 million tons (Reilly et al., 2004) but given potential and observed recovery rates of cetacean species the past four decades (Zerbini et al., 2019), this consumption estimate may be outdated. Subsequently, recovering populations of these major krill consumers may exert significant top‐down pressure on krill, with potential competitive consequences for other predators such as penguins, none of which are currently considered in a management context by CCAMLR.

The humpback whale (*Megaptera novaeangliae*) is a major krill consuming predator in the Southern Ocean. Most individuals migrate annually from low‐latitude breeding grounds to high‐latitude feeding grounds, where they are assumed to consume 83% (Lockyer et al., 1981) of their annual caloric intake during their summer foraging period. Contemporaneous estimates of up to 7000 humpback whales in the northern Antarctic peninsula during summer are now two decades old (Hedley et al., 2001), and were estimated to consume approximately 417,000–806,000 tons of krill biomass in the Antarctic Peninsula alone (Reilly et al., 2004). A recent study of humpback whale reproductive success indicated that numbers in the WAP may be increasing (Pallin et al., 2018), suggesting prolonged recovery from historical harvesting. This is in line with recent population assessments, which indicate that populations in both the western South Atlantic and the eastern South Pacific have recovered to ca. 90% of pre‐exploitation levels (Johnston et al., 2020; Zerbini et al., 2019).

Cetacean abundance is traditionally estimated from line transect surveys using distance sampling as the general analytical framework (Buckland et al., 2005). During line transect surveys, whales are counted along systematic predetermined transects, arranged across the study region to minimize sampling bias in coverage. Distance sampling is then used to estimate a detection probability function that can be applied to estimate whale density (number km^−2^ or nm^−2^) and subsequently estimating the total abundance of whales in the area of interest by simply multiplying the density by the area. The latter assumes that the survey covers representative areas of the entire region (Buckland et al., 2005) and is generally referred to as a “design‐based” approach (Barry & Welsh, 2001). Density Surface Modelling (DSM) is a two‐stage approach for estimating spatially varying density from distance‐sampling data (Miller et al., 2013), and is an appropriate methodology to overcome the assumptions implicit to design‐based approaches. DSM explores potential relationships between animal presence and environmental covariates (Friedlaender et al., 2006, 2011; Herr et al., 2016) to provide potentially more ecologically reliable estimates of abundance throughout a wider area around the survey transects. Extending DSM further to incorporate presence‐absence data with abundance responses to covariates has led to the development of Density Surface Hurdle Models (DSHMs; Franchini et al., 2020), with both DSM and DSHM commonly referred to as “model‐based” approaches.

Optimal line‐transect surveys typically use Research Vessels fitted with double sighting platforms, following predetermined systematic transects arranged across the study region to ensure unbiased sampling coverage (Buckland et al., 2005). However, this requires dedicated vessel and personnel, a sizeable economic burden that makes such surveys costly and, as a result, they are only carried out relatively infrequently. Thus, design‐based surveys of cetaceans tend not to provide information on the spatiotemporal aspects of cetacean abundance and distribution within a season. Given that most cetaceans species perform major seasonal migrations, from lower latitude breeding grounds to higher latitude feeding grounds, they are likely to be neither temporally or spatially stationary, and thus presumably will affect the predator–prey and predator–fishery interactions in both dimensions.

The International Association of Antarctic Tour Operators (IAATO) is the organization responsible for coordinating the use of Antarctica as a tourist destination. In the past two decades, commercially driven tour operators have increased ten‐fold in the Antarctic Peninsula (Bender et al., 2016), and as a consequence provides infrastructure suitable for supporting scientific research, for example as transport between monitoring stations or as opportunistic observation vessels. The use of tourist vessels as surveying platforms not only represents a fraction of the cost of research vessels, but also provides the considerable advantage of following highly repeatable transects multiple times throughout the season. These transects, however, are neither completely systematic nor completely random and lack the same systematic coverage obtained by dedicated research vessels. To address this, model‐based methods (Franchini et al., 2020; Miller et al., 2013) have been developed and become the preferred tool (Williams et al., 2006), compared to traditional methods (Buckland et al., 2005) for assessing cetacean abundance from nonrandom survey data.

Developing EBM for the krill fishery requires understanding and mitigating the risks to nontarget species, both through direct and indirect competitive interactions. Given the poor understanding of the spatiotemporal dynamics in humpback whale abundance and distribution in the WAP area, we used Antarctic cruise vessels as platforms of opportunity to undertake repeated line transect surveys of humpback whales across the Bransfield Strait to develop time‐evolving abundance and distribution estimates throughout the summer season. While our overarching goal was to assess the suitability of platforms of opportunity as cetacean monitoring platforms, our specific goals were to estimate intra‐season variation in (1) humpback whale density and abundance, (2) distribution in relation to environmental drivers, (3) calculate temporally discrete krill consumption estimates, and (4) identify temporal overlap between key ecosystem components. Finally, we draw together our spatiotemporally resolved consumption estimates, with the assumption that whales migrate out of the area in the same way they arrived, into a conceptual model of the overlap in space and time between humpback whales, centrally foraging penguins, and the fishery.

## 2 MATERIALS AND METHODS

### 2.1 Data collection

Marine mammals and birds were counted by Marine Mammal and Seabird Observers (MMSO’s) onboard two IAATO tourist cruise vessels (*MS Midnatsol* and *MS Fram*) along transects in the Scotia Sea and WAP in the period 24 November 2019 to 24 January 2020 (Figure 1). A total of 2 282 nautical miles (nm) were covered or surveyed during 35 days of observational effort in the study area (Table 1). The study area polygon was constructed to coincide with the main area of interest of the regional krill fishery. For analytical purposes, the five trips were grouped into three temporal windows. The first and second trips by *MS Midnatsol* were grouped to “C1,” in order to ensure a sufficient number of sightings for analysis (Buckland et al., 2015). The third *MS Midnatsol* trip and first *MS Fram* trip were grouped to “C2” due to a temporal overlap, while the final cruise of *MS Fram* represents “C3.”

**TABLE 1:** List of the three cruises and five trips with observer effort; M1‐3 represent the three trips on MS Midnatsol, and F1‐2 represent the two trips onboard MS Fram; Cruise C1 (25.11.19–12.12.19), C2 (16–27.12.29) and C3 (12–18.01.20) is the identification of some combined trips for analysis; along with their respective transect length in nautical miles (nm); SA (%) is the area coverage as a percentage of the survey area; Obs(ind; *n*) is the number of observations and number of individuals in parenthesis; encounter rate is measured in observations per nautical mile

Cruise	Trip	Dates in the SA	Effort (nm)	SA (%)	Obs (ind; *n*)	Encounter rate (obs nm^−1^)
C1	M1	25–30.11.19	231.8	0.8	14 (18)	0.07
	M2	5–12.12.19	343.9	1.2	49 (94)	0.27
C2	M3	16–26.12.19	692.8	2.4	125 (203)	0.29
	F1	22–27.12.19	486.8	1.7	72 (132)	0.27
C3	F2	12–18.01.20	527.4	1.9	67 (112)	0.21

On‐effort periods lasted from approximately 05:00 to 22:00 local time and consisted of two or more observers, one as the dedicated recorder and the other(s) as observer(s). Two or more team members were typically stationed on effort, each dedicated as observer or recorder, but in areas of low density of animals, only one member on effort serving as both observer and recorder. During food breaks and necessary rests, effort was either maintained by one observer or ended when no observer was on duty. To the extent possible, these periods were timed to coincide with periods of low‐density animal sightings to minimize the potential of missed sightings. Effort was halted in difficult sighting conditions (Beaufort Sea State >5, or visibility ≲300 m) until conditions improved.

A dedicated Dell Precision 5520 laptop computer was used for logging effort and sightings in the program Logger 2010 (Gillespie et al., 2010), with a Microsoft Access (Microsoft 365 MSO, version 16.0.13328.20334) database back‐end. A Globalsat USB GPS receiver, connected to the laptop computer, recorded coordinates every 10 s, registering location, speed, heading, and time and date. Effort data forms were manually updated in the Logger software for every start and end time of effort activities, changes in weather conditions, observer rotations, and approximately every 30 min, all of which included general environmental conditions and observer roles.

Dedicated observations were carried out from the navigation bridge of each vessel, covering either the port or starboard forward quarter (i.e., 0**°–**270**°** for port and 0**°–**90**°** for starboard, relative to the ship bearing) by scanning for cues using the naked eye, followed by binocular confirmation. Cetaceans were mostly spotted by their blows, but sometimes also through other cues such as breaching or fluking. Immediately following the sighted cue, the recorder would measure radial distance either using a distance stick (Todd et al., 2015) or binoculars (Opticron Marine PS II 7x50/C with integrated distance reticles). Sighting distance was similar for the two ships; bridge height was 13.5 m for *MS Midnatsol* and 13.2 m for *MS Fram*, yielding distances to the horizon of 8.25 and 8.17 nm for the two vessels, respectively, using an average eye height of 1.7 m. The radial angle was measured using an angle board. Radial distance, angle (degrees), and eye height were later used to calculate the perpendicular distance of an animal from the transect line. Additional variables were recorded, such as species, group size, swim direction, and behavior. Photographs of animals were attempted if conditions allowed, but they were not prioritized. Species identifications were based on the shape of the dorsal fin, blow shape and frequency, and species‐specific behavior based on relevant literature (Shirihai, 2006, 2008). In the case of species uncertainty, observations were placed in general categories, such as “large baleen whale” or “like Antarctic minke.”

### 2.2 Data preparation and analysis

The density and abundance of humpback whales were estimated using model‐based density surface hurdle modeling (DSHM; Franchini et al., 2020). Model‐based estimates were achieved by fitting a *detection function* (the probability of animal detection as a function of perpendicular distance) followed by fitting generalized additive models (GAMs; Wood et al., 2016) to explore the relationship of animal presence and abundance to environmental covariates. This relationship was then used to predict animal density in the study area limited by the range of observed environmental values fitted in the GAM (Franchini et al., 2020; Kosicki, 2020; Mannocci et al., 2014). In addition, standard distance sampling estimates were also calculated, which can be found in the Appendix S1.

The *detection function* was fitted to the data using the combined observations from all cruises (C1, C2, and C3) in the study area for best representation of the overall detection probability. To find the best fitting detection function, multiple candidate models were tested. Key functions include half‐normal (HN), hazard rate (HR), and uniform (U; Buckland et al., 2015). Additionally, candidate models included cosine and Hermite polynomial series expansion to find the best fit. Various covariates that may affect sightings probability were also included, such as group size, observer bias, and weather conditions. To reduce the effect of outliers when fitting the detection function, the distance data were truncated at 15% to improve model fit and remove observational outliers (Buckland et al., 2015). Model selection was based on Akaike Information Criterion (AIC; Sakamoto et al., 1986), goodness‐of‐fit, and Cramér‐von Mises test (Darling, 1957).

Model‐based results derived using DSHM protocols (Franchini & Blight, 2020; Franchini et al., 2020) were calculated as the sum of the predicted densities throughout the study area. The DSHM’s, which comprise a binomial presence‐absence (PA) sub‐model and a zero‐inflated count sub‐model (e.g., Poisson, negative binomial) for modeling the number of animals detected conditional on presence (AB), were fitted to the following eight habitat covariates: bathymetric depth and slope; sea surface temperature (SST) and SST gradient (TG); finite‐size Lyapunov exponents (FSLE); distance to nearest coastline (DC). These abiotic habitat covariates were considered proxies or cues for krill distribution. The total number of whales in the study area was calculated by integrating number of predicted whales in the prediction grid, that is, the study area. The goodness‐of‐fit of the DSHM’s was assessed through examination of the QQ plot, and inspection of residuals with respect to distribution, fitted values, and linear predictor. Detailed descriptions of the density and abundance estimation using distance sampling and DSHM, habitat covariates and validations statistics can be found in the Appendix S1.

Data were processed and handled using R Studio (RStudio Team, 2020). Detection function and design‐based distance sampling abundance and density estimates were estimated using the “Distance” package (Miller et al., 2019), while model‐based abundance and distribution estimates were obtained using the “dshm” package implementing the DSHM (Franchini & Blight, 2020).

### 2.3 Prey consumption and temporal overlap

Daily per capita consumption estimates were derived using both traditional estimates (Kleiber, 1961; Reilly et al., 2004) and more recent literature (Acevedo & Urbán, 2021), assessing the summer feeding season of humpback whales. Calculating traditional estimates, we used a daily individual consumption range of 390–874 kg (Reilly et al., 2004). For contemporary estimates (Acevedo & Urbán, 2021), we used a daily individual consumption estimate of 2263 kg (95% CI: 1800–2727). Both estimates are derived using identical values for the energy content of Antarctic krill (1100 kcal kg^−1^; Clarke, 1980).

To estimate temporal trends, we fit our data points to a sigmoid curve representing animal arrival to the feeding ground and defined the beginning of the feeding season as when 50% of the population had arrived, based on our maximum predicted abundance. One hundred and twenty days (Lockyer et al., 1981) later, the sigmoid curve was mirrored, assuming that humpback whales leave the area at the same rate as they arrived. To calculate seasonal consumption, we used the mean daily abundance, and scaled it with daily individual consumption estimates from our traditional (Reilly et al., 2004) and contemporary (Acevedo & Urbán, 2021) sources and multiplying this to match the 120‐day feeding season assumption.

## 3 RESULTS

**FIGURE 2:**
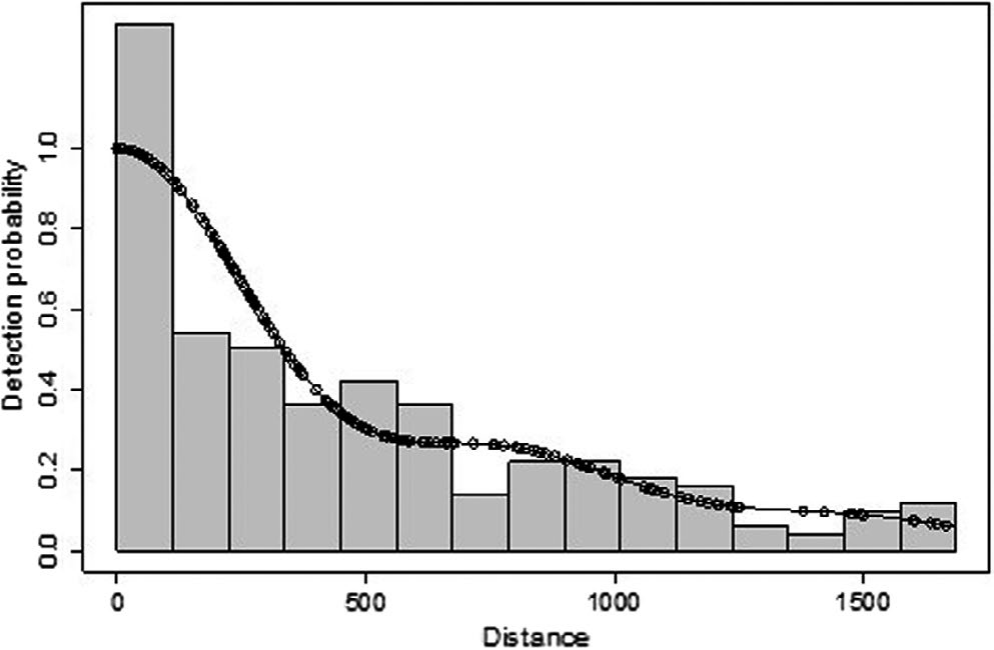
Detection function showing the detection probability of a humpback whale as a function of distance (m)

Mean transect distance per vessel was 457 nm with a range of 232–693 nm. Encounter rates increased significantly from the first trip by *MS Midnatsol* with 0.07 individuals nm^−1^ (14 sightings) in late November to the latter four trips: the second *MS Midnatsol* trip with 0.27 individuals nm^−1^ (49 sightings) in early December; the third *MS Midnatsol* trip with 0.29 individuals nm^−1^ (125 sightings) in late December; the first *MS Fram* trip with 0.27 individuals nm^−1^ (72 sightings) in late December; the second *MS Fram* trip with 0.21 individuals nm^−1^ (67 sightings) in mid‐January (Table 1). The baseline (i.e., no covariates) half‐normal detection function model with cosine adjustment terms was the best model for our observations (Figure 2). Truncation distance was set to exclude 15% of the farthest observations (1685 m) to remove observational outliers (Buckland et al., 2015).

### 3.1 Hurdle models

**FIGURE 3:**
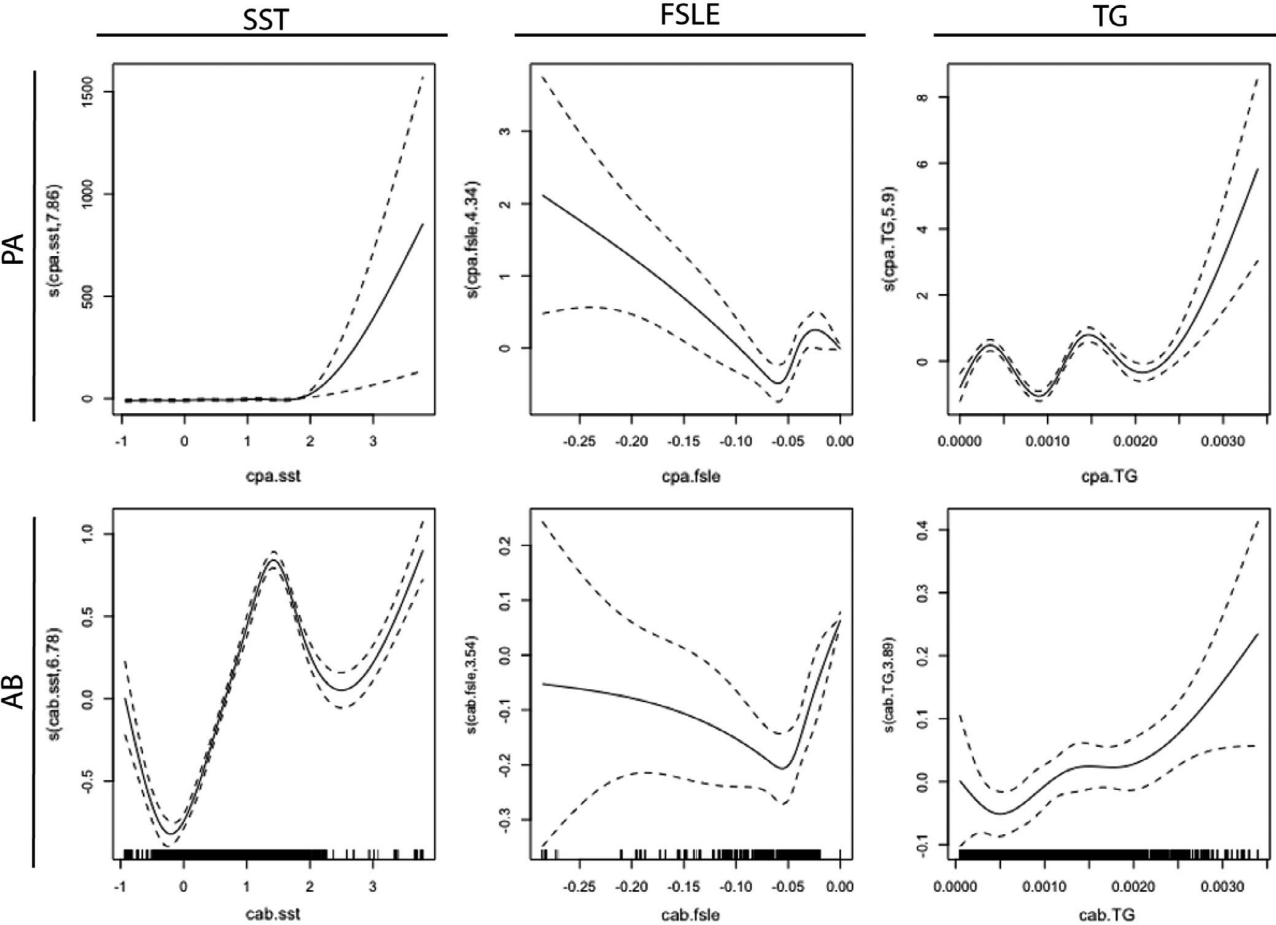
Fitted smooth terms of three habitat covariates: sea surface temperature (SST); FSLE, finite size Lyapunov exponents (FSLE); SST gradient (TG) for presence‐absence (PA) and abundance (AB) of humpback whales

The best submodels (both the binomial PA and zero‐inflated Poisson AB), which included surface temperature (SST), temperature gradient (TG), and oceanographic coherent structures (FSLE) and the baseline detection function, explained 30.5 and 33.7% of the overall deviance for PA and AB, respectively (Figure 3). A complete list of all models run for the DSHM along with deviance explained and ΔAIC is shown in Appendix S1: Tables S1 and S2 for PA and AB submodels, respectively.

The presence of whales was low irrespective of sea surface temperature variations below 2°C but increased with increasing temperatures above 2°C (Figure 3). Humpback whales showed an increasing affinity to FSLE values of −0.10 to −0.25 but showed no distinct trends in values closer to zero. Finally, our data showed a nonlinear increase in the probability of presence toward higher temperature gradients. When humpbacks were present, their abundance was positively correlated to sea surface temperature, showing a rapid increase with increasing temperatures, with a peak at ~1.5°C. The species also showed a nonlinear increasing affinity toward higher TG areas, while the response to changes in FSLE was weaker than for the other two covariates. Additional test statistics can be found in Appendix S1.

### 3.2 Predicted distribution

**FIGURE 4:**
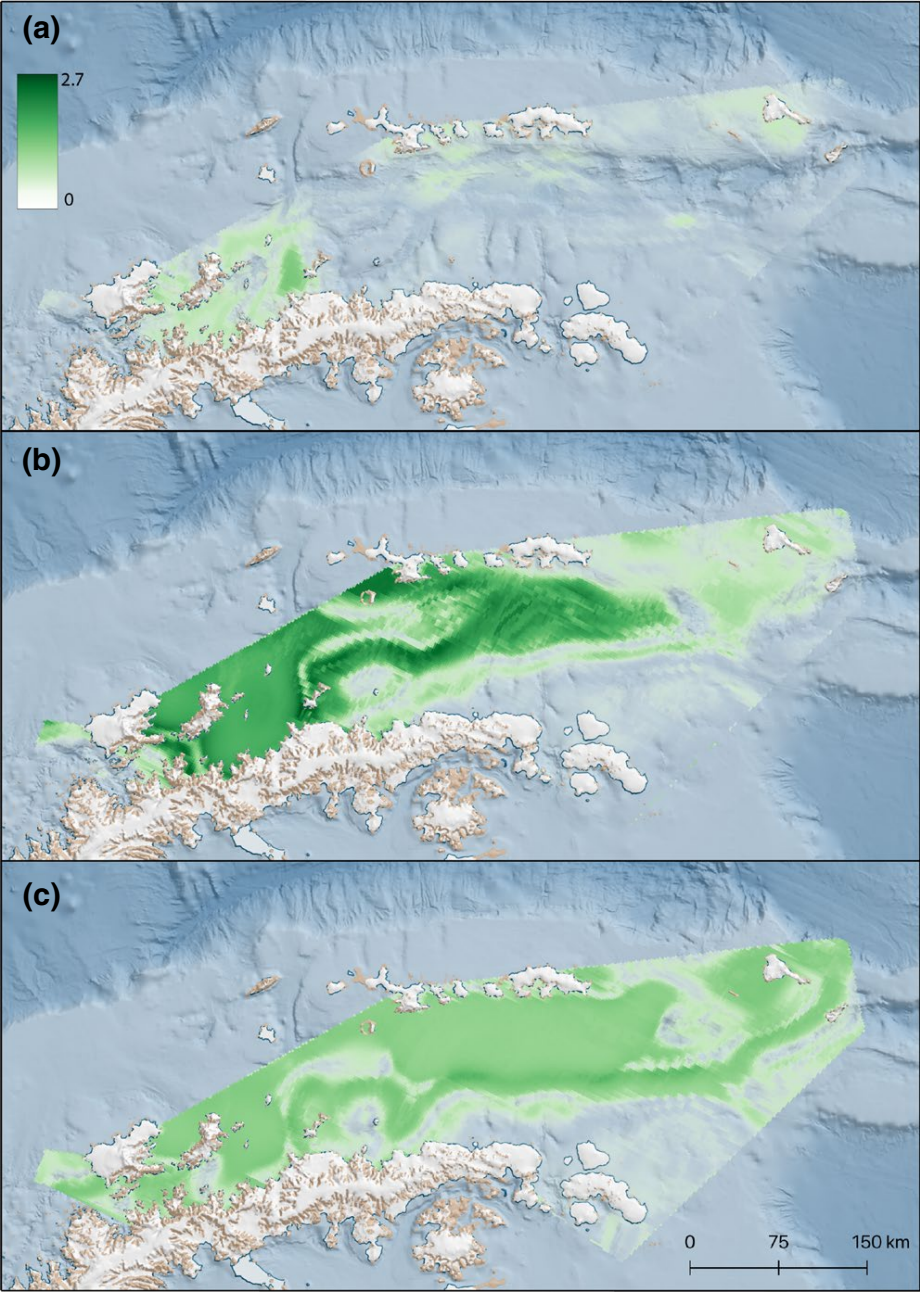
Predicted humpback whale distributions for C1 (a; 25.11.19–12.12.19), C2 (b; 16–27.12.19), and C3 (c; 12–18.01.20) in the Gerlache and Bransfield Straits. Darker colors indicate higher densities of humpback whales, ranging from 0 to 2.7 individuals nm^−2^

Predicted humpback whale densities across the prediction grid for all three cruises, obtained from the hurdle model, are plotted in Figure 4a–c. Density predictions of humpback whales from the DSHM ranged from 0.0006 to 1.362 animals nm^−2^ in C1, with highest values observed around the northern Gerlache Strait, and lowest values southeast of the South Shetland Islands, and south of Elephant Island (Figure 4a). C2 densities ranged from 0.0003 to 2.625 animals nm^−2^, with a broader distribution in the Gerlache Strait and central Bransfield Strait (Figure 4b). C3 densities ranged from 0.0198 to 1.627 animals nm^−2^, with a relatively more expansive, but less dense, distribution in the central and northern Bransfield Strait and the Gerlache Strait (Figure 4c). See Appendix S1: Figure S3 for plots showing the spatial covariate fields across the prediction grid for all 3 cruises.

### 3.3 Abundance and density estimates

Total abundance (i.e., the sum of predicted densities per grid cell) deriving from the DSHMs had a mean of 13,964 individuals, ranging from 4124 to 19,107 individuals throughout the study period (Table 2). Results from C1 (early December) were the lowest at 4124 individuals, while the abundances from C2 and C3 (late December and Mid‐January, respectively), were notably similar at 19,107 and 18,662. Overall, the trend in abundance indicates a significant increase throughout December reaching a plateau at the end of the month.

**TABLE 2:** Density and abundance of humpback whales in the Western Antarctic Peninsula during three cruises C1 (25.11.19–12.12.19), C2 (16–27.12.29) and C3 (12–18.01.20) using the Density Surface Hurdle Modelling (DSHM) method

Cruise	Relative abundance ($\hat{N}$)	Mean density (ind nm^−2^)	Density range (min–max ind nm^−2^)
C1	4124	0.185	0.0006–1.326
C2	19,107	0.860	0.0003–2.625
C3	18,662	0.840	0.0198–1.627

Note

As the relative abundance derived from DSHM predictions are the sum of predicted densities, 95% confidence intervals are not available.

### 3.4 Consumption estimates

Mean daily krill consumption for humpback whales throughout the study period was estimated to be 12,205 kg by traditional sources (Reilly et al., 2004) and 31,601 kg by contemporary sources (Acevedo & Urbán, 2021; Table 3). Intuitively, seasonal variation of consumption is parallel to the seasonal trends in abundance mentioned above. However, overall seasonal consumption was estimated to range from 1.4 to 3.7 million tons, respective of literature.

**TABLE 3:** Daily humpback whale consumption estimates based on abundance estimates derived from Density Surface Hurdle Modelling (DSHM), following daily consumption estimates by traditional and contemporary literature

Cruise	Relative abundance ($\hat{N}$)	Traditional (Reilly et al., 2004)		Contemporary (Acevedo & Urbán, 2021)	
		Lower (t)	Upper (t)	Mean (t)	95% CI
C1	4124	1608	3604	9333	7423–11,246
C2	19,107	7451	16,700	43,239	34,393–52,105
C3	18,662	7278	16,311	42,232	33,592–50,891

Note

Cruises are combinations of some trips defined as C1 (25.11.19–12.12.19), C2 (16–27.12.29) and C3 (12–18.01.20). Traditional estimates use the lower and upper range of consumption from Reilly et al. (2004), while contemporary estimates show the mean and 95% confidence interval (CI) from Acevedo and Urbán (2021). Consumption estimates shown in metric tons (10^3^ kg; t).

## 4 DISCUSSION

We provide the first assessment of within‐season spatial and temporal trends of humpback whale distribution in the WAP (Andrews‐Goff et al., 2018; Weinstein & Friedlaender, 2017), and provide an update to Hedley et al. (2001) with abundance estimates that are in line with projected postharvest recovery rates. While humpback whales are present in the northern Gerlache strait in late November, our results indicate a large‐scale migration into the Bransfield Strait occurs throughout December and January. During early summer, humpback whales appear to aggregate in the northern Gerlache Strait, expanding later into the Bransfield Strait as abundances increase. These findings agree well with telemetry studies indicating the Gerlache and southern Bransfield Straits as hotspots throughout the feeding season (Weinstein & Friedlaender, 2017). During recent years, the fishery has operated in the northern Gerlache Strait during the summer (Krüger, 2019; Santa Cruz et al., 2018); our study highlights that this area is also important for humpback whales arriving early in the summer and remains an exploited area throughout their feeding season. Identifying the spatiotemporal variation in habitat use and important geographical areas for this species in its feeding season provides an insight into interactions between humpback whales and other krill predators, including both penguins and the fishery (Trivelpiece et al., 2011).

### 4.1 Spatiotemporal variation in humpback densities

Using oceanographic covariates as proxies or cues for Antarctic krill to predict distribution of baleen whales such as minke (*Balaenoptera bonaerensis*; Friedlaender et al., 2006, Ainley et al., 2012), fin (*Balaenoptera physalus*; Herr et al., 2016) and humpback whales (Friedlaender et al., 2006, 2011; Herr et al., 2016; Williams et al., 2006) is common practice. Nutrient‐rich Circumpolar Deep Water (Prézelin et al., 2000) heavily influences the Gerlache and Bransfield Straits (Ballerini et al., 2014), entering the study area from the west and subsequently mixing with shelf water to create Transitional Zonal Waters with Bellingshausen Sea influence (TBW; García et al., 2002). Our results indicate that humpback whales tend to be more abundant in this warmer, fresher water mass, compared to the colder, saltier water mass with Weddell Sea influence entering the study area from the northeastern part of the Bransfield Strait (García et al., 2002), indicating higher densities of krill in these waters. The presence and abundance of humpback whales declined with increasing FSLE until a lower threshold (~−0.5) suggesting that humpback whales prefer areas of higher particle retention. Such regions are known to attract predators, given that they tend to be areas with higher retention of primary productivity, such as frontal‐ and eddie systems, both of which are found in the Gerlache and Bransfield Straits (Anadón & Estrada, 2002; Sangrà et al., 2011). Combined, our study underlines the importance of western TBW influence and submesoscale hydrographic structures.

The hurdle models predicted an overall low density early in the season, with hotspots mostly centered on the northern Gerlache Strait, followed by high aggregations of humpback whales in the northern Gerlache Strait and southern‐ and central Bransfield Strait during the “mid‐season,” ensued by a dispersion of animals throughout the study area towards the end of January. Our findings emphasize the importance of the northern Gerlache and western Bransfield Straits as a hotspot for foraging humpback whales through the first half of the summer feeding season. These results align well with regional Antarctic krill spawning events taking place in the Gerlache Strait in late December (Huntley & Brinton, 1991), and humpback whales taking advantage of the local spawning hotspots and nursery areas for immature krill (Cleary et al., 2016; Perry et al., 2019), exhibiting size‐dependent predation, preferring smaller size ranges of krill (≤34 mm; Friedlaender et al., 2008, 2009; Santora et al., 2010). Future work should examine whether these results are consistent over time.

Inherently, cruise vessels will not have the same spatial coverage as dedicated research vessels. Model‐based approaches such as two‐part DSHMs work to overcome this by using habitat models to predict distribution throughout the area of interest (Gowan & Ortega‐Ortiz, 2014; Scott‐Hayward et al., 2015; Waggitt et al., 2020; Warwick‐Evans et al., 2021). This methodology is very beneficial for spatial management analyses in remote areas using limited datasets but does include more uncertainty compared to more traditional methods (Buckland et al., 2005). Comparing our transects (Figure 1), C1 has the least coverage, mostly sailing in the western parts of the study area, while the C2 and C3 transects also cover northern and eastern areas. Thus, C1 predictions may have a higher level of uncertainty, specifically in eastern areas. However, these predictions were made within the confines of observed environmental values (Franchini et al., 2020). Further, the insight gained in the spatiotemporal aspect of the ecosystem agrees well with previous studies using similar analyses (Friedlaender et al., 2021; Herr et al., 2016; Williams et al., 2006), and will be useful in spatial management and conservation efforts.

### 4.2 Abundance and recovery

The Southern Ocean populations of humpback whales are increasing significantly (Pallin et al., 2018; Tulloch et al., 2018), some of which have recovered to near pre‐exploitation levels (Zerbini et al., 2019). Comparable abundance estimates in the area stem from Hedley et al. (2001), concluding with an abundance of approximately 7000 individuals, which is ca. 12,000 lower than the highest abundance in this study (19,107 individuals). This corresponds to an annual population growth of 5.1%, which agrees with the estimates of between 3.1% and 11.8% found elsewhere in the literature (Stevick et al., 2003; Zerbini et al., 2010).

Marine ecosystems are highly dynamic, and this region is no exception. The WAP ecosystem displays significant seasonal dynamics in primary production, with a consequent high variability in the abundance and distribution of upper trophic predators such as migratory baleen whales. Some Antarctic feeding grounds are used by several subpopulations of humpback whales, such as the northern Antarctic Peninsula (Albertson et al., 2018; Dalla Rosa et al., 2008; Robbins et al., 2011), and these areas may be experiencing compounding predatory pressure on krill with a collective subpopulation growth rather than one singular. As a consequence, abundance estimates and population growth will be higher in the feeding grounds compared to the breeding grounds. This has implications for management; when managing a fishery in the feeding ground, data from breeding grounds may give an insight as to the status of upper trophic competition but will underestimate the abundance and growth rate of the population in the management area.

The dynamic nature of these animals underlines the need for both fine‐ and coarse‐scale data collection on krill and its predators at regular intervals in future in order to appropriately determine the degree and significance of predatory pressure. Our approach, using cruise vessels as observation platform, supports earlier work by Williams et al. (2006) in identifying a cost‐effective mechanism to monitor marine mammals and seabirds.

### 4.3 Potential interactions between humpback whales and fisheries

With the contraction of the Antarctic krill fishery around the Antarctic Peninsula and Scotia Sea over recent decades, there is an increasing concern over interactions with and impact of the fishery and other upper trophic predators such as penguins and seals. In the context of direct competition, both spatial and temporal overlap between the components need to be in place (Hinke et al., 2017). Comparing our results spatially with average fishing activity, the past decade (Appendix S1: Figure S4), fishing activity and humpback whales overlap along the frontal systems of the central Bransfield Strait and the northeast Gerlache Strait.

### 4.4 Consumption estimates and temporal overlap

Traditional estimates used to quantify the energetic needs of animals have been based on the Kleiber Equation (Kleiber, 1961), addressing basal metabolic rate. However, recent studies have incorporated detailed physical and biological parameters by high‐resolution using high‐resolution tagging of humpback whales and in situ hydroacoustic prey‐field density estimates to more completely assess the caloric requirements of cetaceans (Acevedo & Urbán, 2021; Smith et al., 2015). Additionally, Acevedo and Urbán (2021) estimated energetic requirements for the Panama/Costa Rica subpopulation, which travel to the Magellan Strait, Chile, for their summer feeding season (Acevedo et al., 2017), a highly comparable group to the Ecuador/southern Colombia subpopulation that migrate to the Antarctic Peninsula over the same period (Stevick et al., 2004).

**FIGURE 5:**
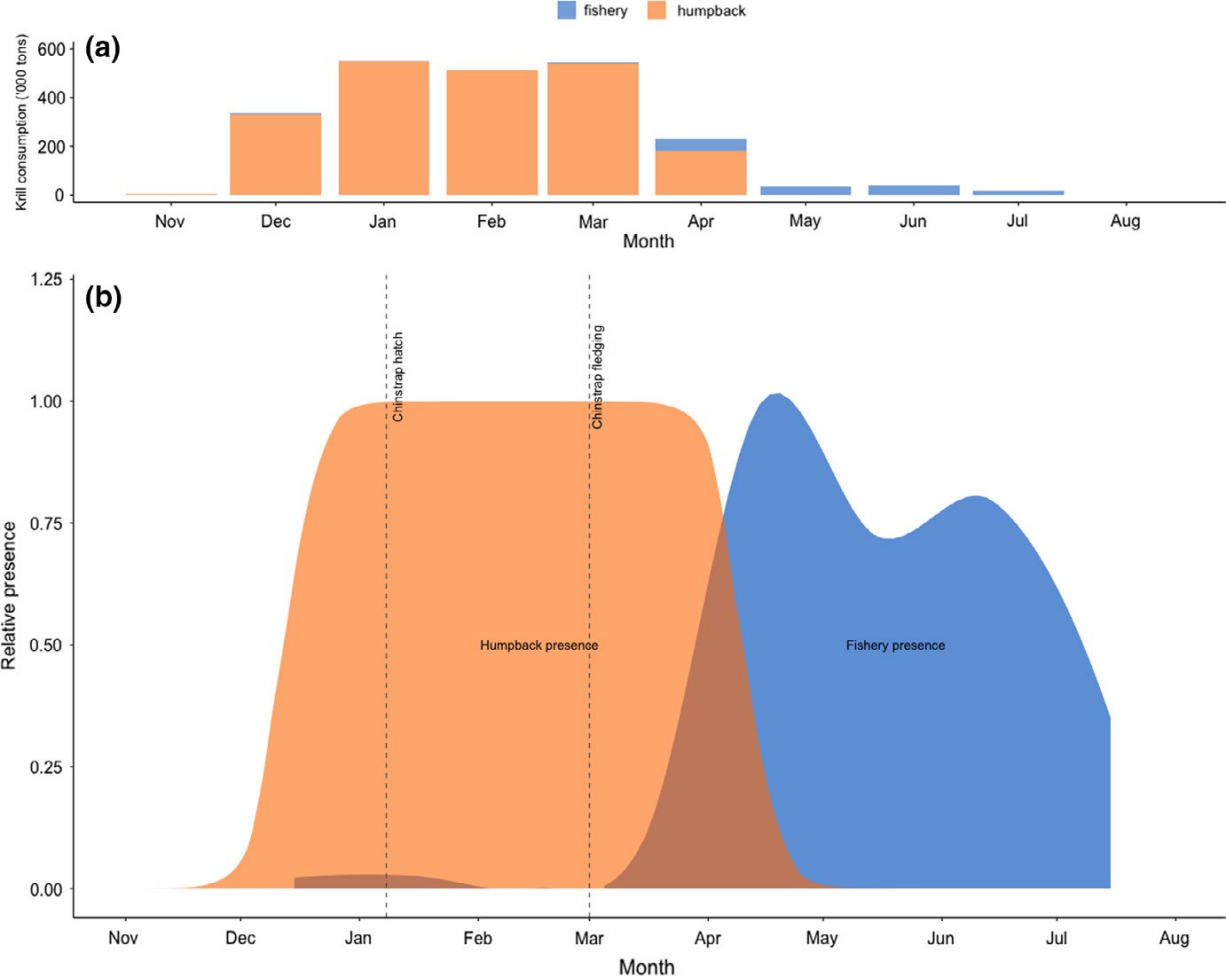
Estimated total krill consumption by humpback whales following traditional literature and fishery catches (a) and the proportion of humpback whales (numbers) and fishery catches in the Gerlache and Bransfield Straits (b). The dotted vertical lines represent the chinstrap penguin hatching and fledging dates

Given the recovery of humpback whales and their spatiotemporal overlap with other krill‐dependent predators, a natural extension of our work is to estimate what competitive pressure is exerted by the former on the latter. For comparison, the annual anthropogenic harvest of krill in the WAP area constitutes a minor fraction (9%) of seasonal krill consumption by humpback whales in this area. Assuming our temporally resolved estimates of habitat use are correct, April is potentially the month with the highest temporal overlap, where anthropogenic krill catches constituted 28% of the humpback whale energy budget (Figure 5a). However, the latter half of their feeding season, presumably lasting from February to April, has yet to be properly quantified at a larger scale. The 120‐day feeding season assumption has been useful but may be outdated. Humpback whale telemetry studies have indicated that movement patterns and departure dates in the WAP may be dictated by sea‐ice cover, with a mean departure date of May 12th and some individuals remaining in the northern WAP until July (Weinstein & Friedlaender, 2017). This highlights the importance of properly quantifying the temporal trends of humpback whales in the WAP, as different demographics may depart the feeding ground at different times, leading to varied interactions between the different life history stages of the species and fishing vessels. In contrast to the limited temporal overlap between humpback whales and the fisheries in the WAP, the overlap between humpback whales and other krill‐dependent predators, such as the chinstrap penguin, which hatch and fledge in the presence of foraging humpback whales, is significant (Figure 5b). This species, among others, has flourished in the absence of large baleen whales (Fraser et al., 1992), but have in recent years been experiencing significant declines in population sizes throughout their range (Dunn et al., 2019; Naveen et al., 2012). While the direct cause of this decline is currently unknown, some literature attributes this deterioration to a combination of climate change, pollution, fishing activity, and habitat loss (Krüger et al., 2020; Trathan et al., 2015). Surely some of these factors may be important, but by excluding important predatory interactions such as predation by humpback whales, there is little hope to unraveling the casual mechanisms behind the declining performance in penguins in the WAP area. Additionally, the Antarctic is at the forefront of climate change effects (Meredith & King, 2005), but the ability to correctly measure the biotic effects of climate change is severely limited without a complete understanding of the ecosystem dynamics. This begs the question of how the ecosystem will respond to an increasing abundance of mass krill consumers, such as the recovering cetacean populations.

## 5 CONCLUSION

Our study demonstrates the utility of platforms of opportunity such as tourist vessels as a viable means to collect data in support of EBM in the WAP area. Platforms of opportunity may potentially be the only logistically and financially feasible approach to gather crucial fine‐scale data regarding cetacean abundance, at least until satellite‐derived abundance estimates (Bamford et al., 2020) are reliable enough to be considered suitable from a management perspective. We have provided some insight of the spatial and temporal distribution of humpback whales in the WAP during the first half of their feeding season and found that the northern Gerlache Strait is an important feeding area for humpback whales, with the majority of animals arriving in late December. Our results suggest limited spatiotemporal overlap between the humpback whales and the Antarctic Krill fishery in the area. However, if the assumed seasonal distribution, in which whales occur in high numbers between mid‐December to late‐April, is correct, then there is potential for interaction between fisheries and humpback whales in April. Future efforts should therefore focus on subsequent months and with regular intervals, to fully quantify the extent of the humpback whale feeding season, as well as other regions with potential for overlap with fishery activity. Our study supports the contention that humpback whale populations are recovering from historical harvesting, that their feeding season directly coincides with critical reproductive dates of chinstrap penguins and that cetaceans are a component of the ecosystem that requires greater consideration in the context of fisheries management in the Antarctic Peninsula.

## CONFLICT OF INTEREST

The authors have no conflict of interest to declare.

## AUTHOR CONTRIBUTIONS

**John Elling Deehr Johannessen:** Conceptualization (equal); Data curation (lead); Formal analysis (lead); Investigation (equal); Methodology (lead); Software (supporting); Validation (lead); Visualization (lead); Writing – original draft (lead); Writing – review & editing (lead). **Andrew Lowther:** Conceptualization (lead); Project administration (lead); Supervision (lead); Writing – original draft (supporting); Writing – review & editing (equal). **Martin Biuw:** Conceptualization (equal); Data curation (equal); Methodology (supporting); Software (equal); Supervision (equal); Writing – original draft (supporting); Writing – review & editing (equal). **Ulf Lindstrøm:** Conceptualization (equal); Supervision (equal); Writing – original draft (supporting); Writing – review & editing (equal). **Victoria Marja Sofia Ollus:** Investigation (equal); Writing – review & editing (supporting). **Kalliopi C. Gkikopoulou:** Investigation (equal); Supervision (equal); Writing – review & editing (supporting). **Lucía Martina Martín López:** Investigation (equal); Supervision (equal); Writing – review & editing (supporting). **Wessel Chris Oosthuizen:** Investigation (equal); Supervision (equal); Writing – review & editing (supporting).

## Supporting information

Figure S1

Figure S2

Figure S3

Figure S4

Figure S5

Appendix S1

## Data Availability

All observational data from the two survey vessels can be found at https://doi.org/10.5061/dryad.rfj6q57c9.
