# Supplementary figures and images for: Intra‐season variations in distribution and abundance of humpback whales in the West Antarctic Peninsula using cruise vessels as opportunistic platforms
Source: Ecol Evol. 2022 Feb 9;12(2):e8571. doi: 10.1002/ece3.8571 (PMC8826076; doi:10.1002/ece3.8571)

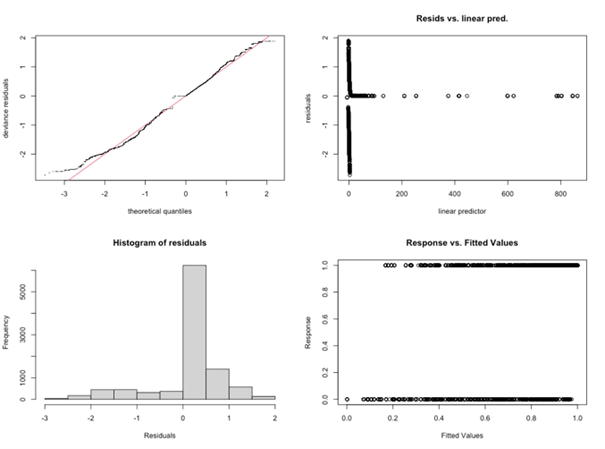

Supplement: Supplementary file 1 — Figure S1 [file ECE3-12-e8571-s003.png]

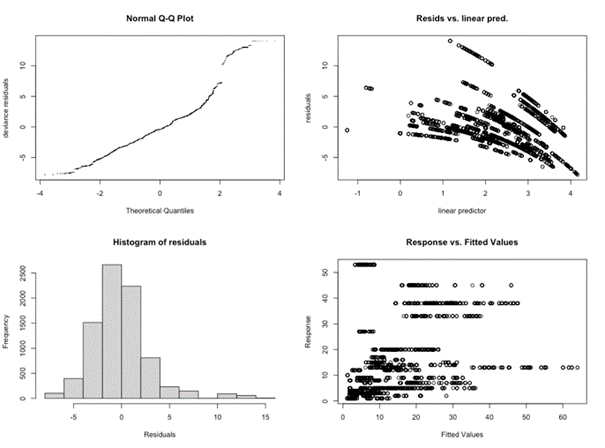

Supplement: Supplementary file 2 — Figure S2 [file ECE3-12-e8571-s002.png]

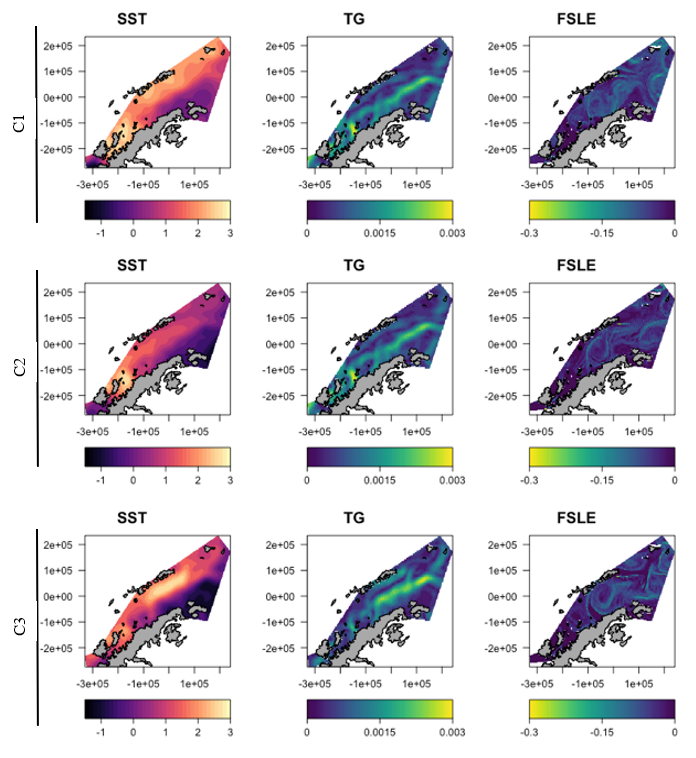

Supplement: Supplementary file 3 — Figure S3 [file ECE3-12-e8571-s006.png]

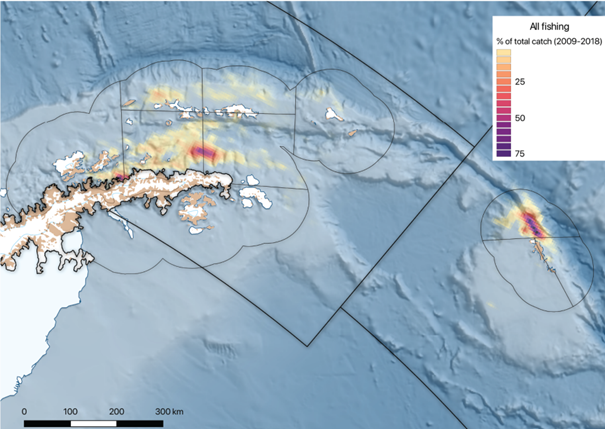

Supplement: Supplementary file 4 — Figure S4 [file ECE3-12-e8571-s001.png]

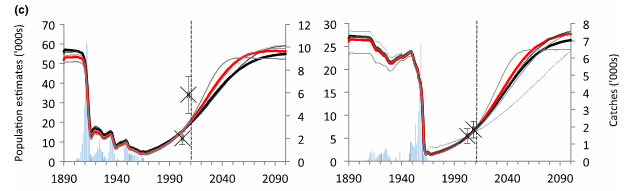

Supplement: Supplementary file 5 — Figure S5 [file ECE3-12-e8571-s005.png]
