# Supplementary material for: Intra‐season variations in distribution and abundance of humpback whales in the West Antarctic Peninsula using cruise vessels as opportunistic platforms
Source: Ecol Evol. 2022 Feb 9;12(2):e8571. doi: 10.1002/ece3.8571 (PMC8826076; doi:10.1002/ece3.8571)
Supplement: Supplementary file 6 — Appendix S1 [file ECE3-12-e8571-s004.docx]

Appendix

Methods

Both design-based and model-based methods use the same on-effort segments and the same fitted animal detection function as the basis, but they use different approaches to estimate density and abundance across the study region. Distance sampling calculates its estimates by using average probability of detection over a given area (transect line length multiplied by perpendicular sighting distance).

*Design-based* results were derived following standard distance sampling protocols (Buckland et al. 2005, 2015), with a slight modification: transect lengths were halved to correct for dedicated observations only being registered in a 90° forward quarter, instead of 180°. The *design-based* density estimates are calculated by dividing the number of sightings with the average detection probability and the surveyed area (transect line length multiplied by perpendicular sighting distance). To get the total abundance of humpback whales in the study area one simply multiplies the density with the size of the study area; this assumes that humpback whales are evenly distributed in the study area.

### *Abundance and density estimates*

Using the fitted detection function and various data from data collection we can calculate density (D̂; abbreviations with "^" mean relative estimates) using distance sampling. This is calculated:

(1)

$$D̂=\frac{n}{2wL\hat{P}_{a}}$$

where *n* is the number of objects detected, *w* is the truncation distance, *L* is the total length of on-effort transect, and $\hat{P}_{a}$is the proportion of animals available for detection (within *w;* Buckland et al. 2015). Relative abundance (N̂) deriving from distance sampling was calculated as:

(2)

$$\hat{N}= D̂ \cdot A$$

using the same approach as density but including the total area (A) of the study area (Buckland et al. 2015).

### *Density surface hurdle modeling*

We use density surface hurdle models (DSHM; Franchini and Blight 2020, Franchini et al. 2020) to predict the spatial distribution, densities and abundances of humpback whales in relation to environmental covariates throughout the study period. Hurdle models are an appropriate means for handling zero-inflated or overdispersed data common in count data (Martin et al. 2005) and in our case the product of two generalized additive models (GAMs); (1) a binomial presence-absence model (PA) and (2) a zero-inflated Poisson abundance model (AB):

(3)

$$p\left( y | \theta, \lambda\right)= \left\{ \begin{aligned} \theta\mathrm{if}y_{n}=0, \mathrm{and} \\ \left( 1-\theta\right)\frac{Poisson (y|\lambda)}{1-PoissonCDF (0|\lambda)}\mathrm{if}y_{n}>0 \end{aligned} \right.$$

where PoissonCDF is the cumulative distribution function of the Poisson distribution. Both these GAMs explore the respective PA and AB response in relation to environmental covariates (Franchini et al. 2020). This modelling approach resulted in predicted densities and distributions across an *a priori* defined prediction grid of 1 nm resolution within the defined study area (Figure 4) with the same oceanographic covariates. Relative abundance ($\hat{N}$) deriving from the fitted DSHMs was found using the sum of densities (D̂) in the modeled prediction grid.

### *Hurdle model calibration and selection*

Both model components (PA and AB) were fitted with a shrinkage version of cubic regression splines to reduce overfitting (Franchini et al. 2020). Models were run and selected based on relative AIC values ($\Delta$AIC), a complete list of the models, their explained deviances, and $\Delta$AIC values are shown in Appendix Table 1 & 2 for PA and AB sub-models, respectively. Knots for each model were selected by trial, avoiding p-values = 0 and number of knots too close to expected degrees of freedom (EDF) and subsequently checked using the 'mgcv' package (Wood et al. 2016). To check for collinearity and concurvity (the "linear" relationship between non-linear variables, where 1 = full concurvity and 0 = no concurvity) of covariates, we ran a model that included all candidate covariates (Appendix Table 3 & 4). Two tests were used to check the GAM sub-model smoothness: (1) Un-Biased Risk Estimator (UBRE) was used to check the smoothness selection score of the binomial PA sub-model, and (2) Generalized (Approximate) Cross-Validation (GCV) was used to check the AB sub-model, in both cases lower values indicate good fit. Goodness of fit was visually assessed by Q-Q and other plots for each sub-model (Appendix Figure 1 & 2 for PA and AB, respectively), and statistically checked using the Kolmogorov-Smirnov test statistic.

### *Oceanographic covariates and prediction grid*

Several environmental covariates, assumed cues for krill availability, were used to explore their relationship to humpback whale counts. Bathymetry data were downloaded using the 'marmap' package (Pante and Simon-Bouhet 2013), querying the National Oceanic and Atmospheric Administration (NOAA) ETOPO1 1 arc-minute global relief model (NOAA National Geophysical Data Center 2009) for the coordinates used in our analysis. The bathymetric slope was calculated using the 'terrain' function in the 'raster' package (Hijmans 2021). Analyzed SST and sea-ice fraction data were downloaded from the Physical Oceanography Distributed Active Archive Center (UKMO 2012), and is a daily sea surface temperature analysis and sea ice analysis produced using optimal interpolation on a 0.054-degree grid. The temperature gradient was found using the same 'terrain' function as bathymetry slope package (Hijmans 2021). Distance to the coast was calculated using the 'geosphere' package (Hijmans 2019), based on our data coordinates and coastal polygons from the Norwegian Polar Institute's Quantarctica package (Matsuoka et al. 2021), for QGIS (v 3.12.1; QGIS.org, 2020). Finite Scale Lyapunov Exponents (FSLE; https://www.aviso.altimetry.fr/) are submesoscale oceanographic features derived from altimetry data. Values in our spatial and temporal frame varied from 0 to ~ -0.28, where lower values represent the retention of passive particles such as phytoplankton at the sea surface for days or even weeks, rather than hours, durations that may be useful to predators (Lowther et al. 2014).

All covariates were extracted for the coordinate and date associated to each observation in the observation data (i.e., data fitted to the DSHMs) while the three prediction grids’ (C1, C2, C3) covariate values were rescaled to fit the grid resolution and consist of mean covariate values for each day of the respective temporal window.

Results

The UBRE test for the binomial PA model yielded a selection score of -0.44, while the GCV test for the zero-inflated Poisson AB model yielded a score of 8.95. Sub-model goodness of fit plots (Appendix figure 2 & 3 for PA and AB, respectively) showed a slightly better fit for the PA sub-model compared to the AB sub-model. Overall, the Kolmogorov-Smirnov (KS) test statistic yielded significant results (KS = 0.176, p-value < 0.01).

Abundance and subsequent consumption estimates deriving from the distance sampling method (Buckland et al. 2005, Miller et al. 2019) are listed in Appendix tables 4 and 5, respectively.

**Table S1** - List of presence-absence (PA) models fitted to observed data including deviance explained (expl_Dev), log likelihood (logLik), second-order Akaike Information Criterion (AICc), and ∆AIC (relative difference in AICc within the models run). Selected model in bold.

| **Presence-Absence (PA)** | | | | |
| --- | --- | --- | --- | --- |
| Model | expl_Dev | logLik | AICc | ∆AIC |
| TG | 8.74 | -4029.84 | 8079.104 | 2374.897 |
| Slope | 0.77 | -4515.64 | 9047.294 | 3343.087 |
| FSLE | 2.55 | -4661.63 | 9342.71 | 3638.503 |
| SST | 24.43 | -4697.31 | 9414.494 | 3710.287 |
| Depth | 6.50 | -5811.63 | 11642.883 | 5938.676 |
| DC | 3.97 | -5886.19 | 11792.175 | 6087.968 |
|  |  |  |  |  |
| SST + TG | 28.69 | -3149.03 | 6335.289 | 631.082 |
| SST + Slope | 26.96 | -3323.86 | 6684.431 | 980.224 |
| TG + FSLE | 13.45 | -3514.44 | 7065.338 | 1361.131 |
| TG + Slope | 10.18 | -3547.42 | 7130.566 | 1426.359 |
| SST + FSLE | 24.80 | -3597.29 | 7232.21 | 1528.003 |
| Depth + TG | 14.85 | -3760.20 | 7557.586 | 1853.379 |
| TG + DC | 13.14 | -3801.58 | 7640.722 | 1936.515 |
| FSLE + Slope | 3.62 | -4040.00 | 8113.096 | 2408.889 |
| Depth + Slope | 7.20 | -4223.26 | 8480.293 | 2776.086 |
| DC + Slope | 5.66 | -4293.07 | 8621.408 | 2917.201 |
| Depth + FSLE | 8.53 | -4375.59 | 8788.188 | 3083.981 |
| FSLE + DC | 6.42 | -4440.14 | 8917.792 | 3213.585 |
| Depth + SST | 27.18 | -4526.36 | 9090.231 | 3386.024 |
| SST + DC | 25.91 | -4541.13 | 9119.8 | 3415.593 |
| Depth + DC | 8.20 | -5626.43 | 11290.199 | 5585.992 |
|  |  |  |  |  |
| **SST + TG + FSLE** | **30.48** | **-2824.53** | **5704.207** | **0** |
| SST + TG + Slope | 27.63 | -2858.48 | 5752.672 | 48.465 |
| SST + FSLE + Slope | 28.90 | -2980.32 | 6015.03 | 310.823 |
| Depth + SST + TG | 32.01 | -3002.11 | 6059.109 | 354.902 |
| SST + TG + DC | 30.54 | -3039.93 | 6133.234 | 429.027 |
| TG + FSLE + Slope | 15.12 | -3178.78 | 6411.414 | 707.207 |
| Depth + SST + Slope | 29.92 | -3189.33 | 6432.008 | 727.801 |
| SST + Slope + DC | 29.11 | -3226.17 | 6505.061 | 800.854 |
| Depth + TG + FSLE | 19.70 | -3260.53 | 6575.419 | 871.212 |
| Depth + TG + Slope | 16.95 | -3280.18 | 6612.166 | 907.959 |
| TG + Slope + DC | 16.94 | -3280.74 | 6616.587 | 912.38 |
| TG + FSLE + DC | 18.19 | -3289.73 | 6634.366 | 930.159 |
| Depth + SST + FSLE | 27.42 | -3472.06 | 6999.2 | 1294.993 |
| SST + FSLE + DC | 26.52 | -3486.21 | 7027.77 | 1323.563 |
| Depth + TG + DC | 17.53 | -3609.19 | 7273.424 | 1569.217 |
| Depth + FSLE + Slope | 9.82 | -3779.85 | 7610.57 | 1906.363 |
| FSLE + Slope + DC | 8.94 | -3816.78 | 7686.456 | 1982.249 |
| Depth + Slope + DC | 10.23 | -4085.25 | 8223.01 | 2518.803 |
| Depth + FSLE + DC | 11.04 | -4220.99 | 8496.996 | 2792.789 |
| Depth + SST + DC | 28.37 | -4390.23 | 8835.575 | 3131.368 |

**Table S2** - List of abundance (AB) models fitted to observed data including deviance explained (expl_Dev), log likelihood (logLik), second-order Akaike Information Criterion (AICc), and ∆AIC (relative difference in AICc within the models run). Selected model in bold.

| **Abundance (AB)** | | | | |
| --- | --- | --- | --- | --- |
| Model | expl_Dev | logLik | AICc | deltaAIC |
| TG | 5.08 | -70604.73 | 141231.3 | 23340.6 |
| Slope | 18.84 | -72623.44 | 145268.8 | 27378.1 |
| FSLE | 0.58 | -74538.74 | 149099 | 31208.3 |
| SST | 1.30 | -75680.73 | 151382 | 33491.3 |
| Depth | 5.15 | -81643.84 | 163309.4 | 45418.7 |
| DC | 3.05 | -82503.55 | 165027 | 47136.3 |
|  |  |  |  |  |
| SST + TG | 19.10 | -62549.70 | 125136.1 | 7245.4 |
| SST + Slope | 19.77 | -63271.10 | 126581.1 | 8690.4 |
| SST + FSLE | 21.51 | -63573.66 | 127186.2 | 9295.5 |
| TG + Slope | 6.13 | -65913.31 | 131866.1 | 13975.4 |
| TG + FSLE | 7.16 | -66816.81 | 133671.7 | 15781 |
| TG + DC | 10.54 | -67054.12 | 134147.7 | 16257 |
| Depth + TG | 9.98 | -67789.26 | 135618.4 | 17727.7 |
| SST + DC | 23.37 | -69201.55 | 138442.7 | 20552 |
| Depth + SST | 21.51 | -70864.15 | 141767.8 | 23877.1 |
| FSLE + Slope | 1.98 | -71617.47 | 143273.3 | 25382.6 |
| Depth + Slope | 4.96 | -71965.00 | 143968.2 | 26077.5 |
| Depth + FSLE | 7.03 | -72250.97 | 144541.5 | 26650.8 |
| DC + Slope | 2.92 | -73164.17 | 146367.8 | 28477.1 |
| FSLE + DC | 3.74 | -73796.28 | 147630.1 | 29739.4 |
| Depth + DC | 8.70 | -78806.30 | 157650.6 | 39759.9 |
|  |  |  |  |  |
| **SST + TG + FSLE** | **33.72** | **-58917.19** | **117890.7** | **0** |
| SST + TG + Slope | 18.54 | -59163.12 | 118365.7 | 475 |
| SST + TG + DC | 24.27 | -59216.02 | 118488.1 | 597.4 |
| SST + FSLE + Slope | 22.14 | -60076.74 | 120210.4 | 2319.7 |
| Depth + SST + TG | 22.70 | -60484.13 | 121025.1 | 3134.4 |
| Depth + SST + Slope | 23.83 | -60893.01 | 121842.1 | 3951.4 |
| SST + Slope + DC | 23.53 | -61063.87 | 122184.6 | 4293.9 |
| SST + FSLE + DC | 25.09 | -61081.86 | 122219.3 | 4328.6 |
| Depth + SST + FSLE | 25.48 | -61194.72 | 122447 | 4556.3 |
| Depth + TG + DC | 18.43 | -62550.61 | 125158.6 | 7267.9 |
| TG + Slope + DC | 11.11 | -63207.29 | 126471.7 | 8581 |
| Depth + TG + Slope | 11.06 | -63233.32 | 126523.8 | 8633.1 |
| TG + FSLE + DC | 12.09 | -63664.46 | 127385.1 | 9494.4 |
| TG + FSLE + Slope | 8.23 | -63692.02 | 127440.9 | 9550.2 |
| Depth + TG + FSLE | 12.66 | -63762.83 | 127583.1 | 9692.4 |
| Depth + SST + DC | 27.99 | -66170.93 | 132399.4 | 14508.7 |
| Depth + FSLE + Slope | 7.31 | -68567.69 | 137190.7 | 19300 |
| Depth + FSLE + DC | 11.07 | -69430.41 | 138916.8 | 21026.1 |
| Depth + Slope + DC | 9.03 | -69579.70 | 139216.2 | 21325.5 |
| FSLE + Slope + DC | 4.31 | -70285.83 | 140627.3 | 22736.6 |

**Table S3** - table showing the concurvity indices for sub-model presence-absence (PA) for each environmental covariate sea surface temperature (SST); FSLE, finite size lyapunov exponents (FSLE); SST gradient (TG).

| Concurvity check PA | | | | |
| --- | --- | --- | --- | --- |
|  | Parameter | SST | TG | FSLE |
| **Worst** | 3.98858e-28 | 0.3796519 | 0.37773374 | 0.13511367 |
| **Observed** | 3.98858e-28 | 0.1773197 | 0.09218982 | 0.10750031 |
| **Estimate** | 3.98858e-28 | 0.1030539 | 0.10351097 | 0.04615805 |

**Table S4** - table showing the concurvity indices for sub-model abundance (AB) for each environmental covariate sea surface temperature (SST); FSLE, finite size lyapunov exponents (FSLE); SST gradient (TG).

| Concurvity check AB | | | | |
| --- | --- | --- | --- | --- |
|  | Parameter | SST | TG | FSLE |
| **Worst** | 2.784855e-28 | 0.34160102 | 0.35894560 | 0.14544183 |
| **Observed** | 2.784855e-28 | 0.07976032 | 0.12155610 | 0.12072565 |
| **Estimate** | 2.784855e-28 | 0.10467141 | 0.09518244 | 0.05794214 |

**Table S5** - Catch (tons) of Antarctic krill in area 48 in 2018. Data collected from CCAMLR Secretariat 2021.

**Table S6** –Density and abundance of humpback whales in the Western Antarctic Peninsula during three cruises C1 (25.11.19 – 12.12.19), C2 (16 – 27.12.29) and C3 (12 – 18.01.20)using the distance sampling (DS) method for comparative reasons. Estimate is total number of humpback whales in the study area; SE is standard error; CV is coefficient of variation; LCL and UCL are lower and upper 2.5 percentiles in the confidence interval; DF is degrees of freedom.

| Cruise | Relative abundance ($\hat{N}$) | SE | CV | LCL | UCL | DF |
| --- | --- | --- | --- | --- | --- | --- |
| **C1** | 11 462 | 3 753 | 0.33 | 5 882 | 22 336 | 19.37 |
| **C2** | 14 142 | 3 439 | 0.24 | 8 700 | 22 987 | 36.90 |
| **C3** | 14 772 | 7 666 | 0.52 | 5 267 | 41 424 | 16.83 |

**Table S7** – Daily humpback whale consumption estimates based on abundance estimates ($\hat{N}$) derived from distance sampling (DS), following daily consumption estimates by Reilly et al. (2004), as upper and lower range of humpback whale consumption, as well as contemporary estimates by Acevedo and Urbán (2021). Consumption estimates shown in metric tons (10^3^ kg; t). Cruises are combinations of some trips defined as C1 (25.11.19 – 12.12.19), C2 (16 – 27.12.29) and C3 (12 – 18.01.20).

|  |  | **Traditional**  **(Reilly et al., 2004)** | | **Contemporary**  **(Acevedo & Urbán, 2021)** | |
| --- | --- | --- | --- | --- | --- |
| **Cruise** | **Abundance (**$\hat{\mathbf{N}}$**)** | **Lower (t)** | **Upper (t)** | **Mean (t)** | **95% CI** |
| **C1** | 11 462 | 4 470 | 10 018 | 25 939 | 20 631 – 31 257 |
| **C2** | 14 142 | 5 515 | 12 360 | 32 003 | 25 456 – 38 365 |
| **C3** | 14 772 | 5 761 | 12 911 | 33 429 | 26 589 – 40 283 |

Appendix Figure Legends

**Figure S1** – Plots showing Q-Q plots, Residuals vs Linear prediction, Histogram of Residuals, and Response vs fitted values for the fitted GAM for the binomial sub-model PA (presence-absence).

**Figure S2** - Plots showing Q-Q plots, Residuals vs Linear prediction, Histogram of Residuals, and Response vs fitted values for the fitted GAM for the zero-inflate Poisson sub-model AB (Abundance).

**Figure S3** – Prediction grids for each cruise C1 (25.11.19 – 12.12.19), C2 (16 – 27.12.29) and C3 (12 – 18.01.20) and environmental covariates: Sea surface temperature (SST), SST gradient (TG), and finite size Lyapunov exponents (FSLE).

**Figure S4** - Map showing the spatial distribution of Antarctic Krill fishery activity in the period 2009 - 2018. Produced by and borrowed from Lowther et al. (2020).

**Figure S5** - Projected humpback whale abundances for the Atlantic/Indian (left) and Pacific (right) populations. From Tulloch et al. (2018).

**Appendix Figure 3** - Oceanographic covariates in prediction grid for all 3 cruises
